# A Review of Volatile Organic Compound Contamination in Post-Industrial Urban Centers: Reproductive Health Implications Using a Detroit Lens

**DOI:** 10.3390/ijerph17238755

**Published:** 2020-11-25

**Authors:** Carol J. Miller, Melissa Runge-Morris, Andrea E. Cassidy-Bushrow, Jennifer K. Straughen, Timothy M. Dittrich, Tracie R. Baker, Michael C. Petriello, Gil Mor, Douglas M. Ruden, Brendan F. O’Leary, Sadaf Teimoori, Chandra M. Tummala, Samantha Heldman, Manisha Agarwal, Katherine Roth, Zhao Yang, Bridget B. Baker

**Affiliations:** 1 Center for Leadership in Environmental Awareness and Research (CLEAR)—Integrative Biosciences Center, Wayne State University, 6135 Woodward Ave, Detroit, MI 48202, USA; ab1421@wayne.edu (C.J.M.); mrungemo@med.wayne.edu (M.R.-M.); acassid1@hfhs.org (A.E.C.-B.); jstraug1@hfhs.org (J.K.S.); gk2840@wayne.edu (T.M.D.); tracie.baker@wayne.edu (T.R.B.); michael.petriello@wayne.edu (M.C.P.); gmor@med.wayne.edu (G.M.); douglas.ruden@gmail.com (D.M.R.); ax9873@wayne.edu (B.F.O.); 2 Department of Civil and Environmental Engineering—College of Engineering, Wayne State University, 5050 Anthony Wayne Drive, Detroit, MI 48202, USA; sadaf.teimoori@wayne.edu (S.T.); ctummala@wayne.edu (C.M.T.); 3 Institute of Environmental Health Sciences—Integrative Biosciences Center, Wayne State University, 6135 Woodward Ave, Detroit, MI 48202, USA; manisha.agarwal@wayne.edu (M.A.); katherine.roth3@gmail.com (K.R.); zhaoyang@med.wayne.edu (Z.Y.); 4 Department of Public Health Sciences, Henry Ford Hospital, 1 Ford Place, Detroit, MI 48202, USA; 5 Department of Pharmacology—School of Medicine, Wayne State University, 540 E. Canfield, Detroit, MI 48202, USA; smheldman@wayne.edu; 6 C.S. Mott Center for Human Growth and Development, Wayne State University, 275 E. Hancock, Detroit, MI 48201, USA; 7 Department of Obstetrics and Gynecology, Wayne State University, 275 E. Hancock, Detroit, MI 48201, USA

**Keywords:** volatile organic compounds, vapor intrusion, adverse birth outcomes, health disparities, remediation, urban

## Abstract

Volatile organic compounds (VOCs) are a group of aromatic or chlorinated organic chemicals commonly found in manufactured products that have high vapor pressure, and thus vaporize readily at room temperature. While airshed VOCs are well studied and have provided insights into public health issues, we suggest that belowground VOCs and the related vapor intrusion process could be equally or even more relevant to public health. The persistence, movement, remediation, and human health implications of subsurface VOCs in urban landscapes remain relatively understudied despite evidence of widespread contamination. This review explores the state of the science of subsurface movement and remediation of VOCs through groundwater and soils, the linkages between these poorly understood contaminant exposure pathways and health outcomes based on research in various animal models, and describes the role of these contaminants in human health, focusing on birth outcomes, notably low birth weight and preterm birth. Finally, this review provides recommendations for future research to address knowledge gaps that are essential for not only tackling health disparities and environmental injustice in post-industrial cities, but also protecting and preserving critical freshwater resources.

## 1. Introduction

Post-industrial urban centers such as Detroit, Michigan (MI), a home of early industrialists including Ford and Dodge [1], are faced not only with the uncertainties of legacy (e.g., lead), but also emerging environmental contaminants, including anthropogenic volatile organic compounds (VOCs). Similar to Detroit, many of the nation’s post-industrial cities border the Great Lakes [2] because of access to immense volumes of freshwater, which proved essential for the power generation and waste stream outlet needs of burgeoning industries. The Great Lakes watershed is an invaluable resource-rich region, harboring 21% of the world’s freshwater resources and supplying the drinking water for more than 30 million people in the bordering states and provinces [3]. Thus, ironically, while the region boasts plentiful freshwater resources, the Great Lakes also hosts many of the most polluted cities in the nation. Economically, MI and its Great Lakes neighbors can be conceptualized as the fulcrum of a much larger mega-economy, which if it were its own country, would have a gross domestic product of more than $6 trillion (USD)—making it the third largest economy in the world [4]. This places Detroit and other Great Lakes border cities in a position of tremendous responsibility to protect and preserve the planet’s freshwater resources for current and future generations.

Detroit has had its share of economic challenges in recent years and is still recovering from the largest municipal bankruptcy filing in U.S. history in 2013. New efforts aimed at urban renewal and revitalization are juxtaposed with a vast landscape of aging urban infrastructure that threatens the quality and safety of the existing built environment. In recognition of the tremendous infrastructure problems throughout MI, the recent American Society of Civil Engineers Infrastructure Report Card (2018) [5] assigned the region a D+ for the health of its infrastructure. Tied to this aging infrastructure, many vulnerable persons living and working in Detroit, which is 80% African American, suffer from both environmental hazards and health disparities. The 2015 lead and Legionella water contamination disaster in Detroit’s smaller sister city, Flint, MI, brought such silent urban public health crises to center stage in the media and the world’s attention [6,7].

VOCs are a group of aromatic or chlorinated organic chemicals that have high vapor pressure, and thus vaporize readily at room temperature. They originate from diverse manufactured products such as building materials, paints, cleaning agents, furnishings, adhesives, combustion materials, floor, and wall coverings. People are exposed to volatile contaminants through dermal contact, inhalation, and ingestion. Prevalence of VOCs in the airshed overlying urban landscapes has been well studied and are especially problematic [8,9], negatively influencing human health [10,11]. Of particular concern in urban areas are persistently poor maternal/child health outcomes. Maternal environmental exposure to the VOCs tetrachloroethylene (PCE) [12] and trichloroethylene (TCE) [13] have specifically been linked to adverse birth outcomes, and prenatal exposure to ambient VOCs has been associated with significantly lower birth weight [14] and higher preterm birth (PTB) rates [15,16]. Detroit has one of the highest PTB rates in the United States (USA) at 14.3%, with Black women experiencing a 57% higher PTB rate compared to all other women in Michigan [17]. Cleveland, Ohio, another Great Lakes border city, is the only city in the U.S. that surpassed Detroit with a PTB rate of 14.5%. Though the causes of this reproductive health crisis in urban environments in the Great Lakes basin are likely multifactorial, environmental chemical exposure coupled with socio-economic health disparities are among the top preventable targets for intervention strategies.

While information about airshed VOCs may provide insights into some public health issues, most people in the U.S. spend at least 90% of their time indoors [18]; thus, we suggest that VOC concentrations arising from contaminated soil or groundwater could be equally or even more relevant to public health issues due to vapor intrusion [19,20,21,22,23]. This is even more relevant considering the new lifestyles during the COVID-19 pandemic, with time inside homes greatly increased [24]. Subsurface intrusion into buildings was first introduced as a consideration on the Hazardous Ranking System in 2018 and human expose to hazardous pollutants via this mechanism can now be used for Superfund designation [25]. Though VOCs and their movement and persistence belowground in urban landscapes remain relatively understudied [26,27], of the 67 Superfund sites on the National Priorities List in the State of Michigan, at least 37 have some type of underground VOC contamination. Many of the sites are either former waste disposal (i.e., landfills) or manufacturing sites, containing some amount of contamination for which many clean-up efforts incorporate soil and groundwater remediation. While originally thought to be confined to these designated Superfund or other highly contaminated sites (i.e., “brownfield sites”), VOCs are now known to be much more widespread across the urban landscape, impacting homes, businesses, schools, and all facets of the built environment in legacy cities including Detroit [28,29,30].

VOCs, especially chlorinated solvents, can persist belowground in soils and shallow aquifers for decades [31,32,33] and travel through the groundwater and subsurface soils [34,35]. VOCs may enter the subsurface via multiple pathways, which will have different spatial distributions depending on the source, e.g., specific point sources such as surface releases (leaking drums and spills), subsurface releases (underground storage tanks), leaking sewer pipes [32,36,37] or potential non-point sources such as stormwater runoff/infiltration [38,39,40,41]. VOCs can volatize as “plumes” from shallow groundwater and contaminated soil into soil vapor and then move into residential and commercial buildings through openings such as foundation/slab cracks, utility lines and piping, and sump pump openings, creating a hidden source of indoor air pollution [12,42,43]. The chemicals broadly referred to as VOCs include a wide variety of characteristics with transport rates in groundwater and soil dependent on compound-specific adsorption, biodegradation, dispersion/diffusion, and volatilization parameters [32]; thus, assessment, containment, modeling, and analysis must acknowledge these differences. While concerns of vapor intrusion exist for all compounds in the VOC class, benzene, PCE, TCE, and vinyl chloride are of particular concern in metropolitan (metro) Detroit [44] where the primary sources of subsurface environmental VOCs are leakage from liquid storage tanks and waste streams from metal processing facilities. Observed concentrations in groundwater and soil in metro Detroit extend up to 7,900,000 ug/kg benzene and 150,000 ug/L PCE, respectively [45]. While typically linked to over 4800 legacy/brownfield sites per the Michigan Department of Environment, Great Lakes, and Energy, some of the most highly publicized cases in metro Detroit are associated with current discharges from active facilities (e.g., the “green goo”) [46]. Detection of belowground VOCs rely on a variety of techniques; all require special attention to limit loss of volatile compounds during the collection process. Various techniques are generally available in state regulatory guidance manuals, such as described in [47].

VOCs are a part of everyday life and the sources of indoor VOCs can be largely dependent on the activities of people inhabiting the building. For example, homes with attached garages and basements can influence indoor air quality by storing VOC-emitting products such as automobiles, paints, and solvents [48,49]. Given the difficulty in assigning sources to VOC compounds, many vapor intrusion guidance documents recommend multiple lines of evidence to determine the extent of VOC vapor intrusion [50,51,52]. Another pathway for VOC plumes to gain access to homes is through basement leaks and flooding, a common and recurrent infrastructure problem in Detroit [53,54], allowing contaminants to insinuate into indoor gas and particulate phases, surface areas, and fabrics [55]. This vapor intrusion process has the potential to place residents or employees of buildings located above or near soils and groundwater contaminated with VOCs at risk for major health problems [12,56]. The VOC concentrations of indoor air resulting from vapor intrusion vary spatially and temporally, with typical variations of several orders of magnitude within several months [57].

There is an urgent and unmet need in the city of Detroit to understand and remediate the consequences of VOC environmental exposure on humans. In this review, we explore the state of the science of subsurface movement and remediation of VOCs, and the linkages between these little-understood contaminant exposure pathways and health consequences based on research in various animal models and in adverse birth outcomes in humans, notably low birth weight and PTB. We seek to better understand how volatile and semi-volatile organic compounds from urban brownfield sites may contaminate the subsurface beneath the built environment and through a combination of liquid and vapor transport, expose and negatively impact humans. Thus, we aim to uncover significant knowledge gaps that limit our ability to explain the complex source-migration-exposure-outcome scenario of VOCs in urban settings, tackle health disparities and environmental injustice in post-industrial cities, and protect and preserve critical freshwater resources.

## 2. VOC Transport Pathways

**Figure 1:**
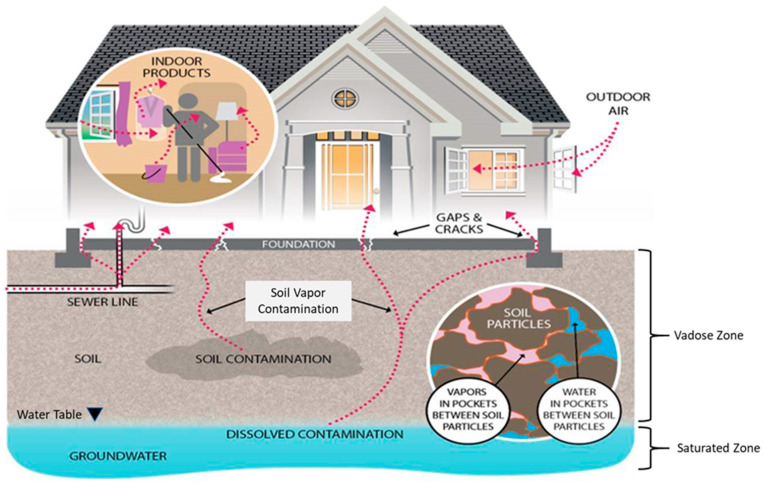
Vapor intrusion pathways [62].

Groundwater provides a conduit for chemical movement through urban systems. Volatile chemicals, such as VOCs, can move from groundwater into the unsaturated zone, or vadose zone, as soil vapor. Soil vapor causes a vapor intrusion concern when volatile chemicals in contaminated soil or groundwater migrate through the subsurface soils and/or preferential pathways and impact the indoor air quality of buildings [58]. In this way, groundwater contributes to potential exposure routes with the groundwater table influencing water and soil vapor movement through the vadose zone [59,60,61]. Figure 1 illustrates the multiple pathways that play a role in subsurface transport of VOCs in residential settings. This section describes shallow urban groundwater movement with the objective of understanding how current and legacy VOC contaminants move in the vadose zone.

**Figure 2:**
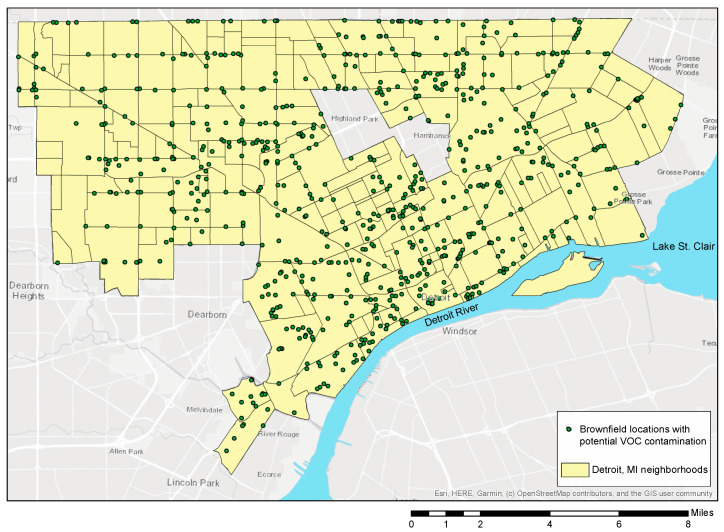
Detroit brownfield locations that can serve as sources of VOCs [68]. Note that Highland Park and Hamtramck are not part of the city of Detroit.

Shallow groundwater has a high risk of contamination from surface spills and buried waste due to the relatively short vertical distance to the water table and small hydraulic retention time [63,64,65]. Areas with shallow groundwater, including coastal southeast MI and Detroit are impacted by additional environmental problems which include direct flooding of houses during storm events and VOC vapor intrusion [53,66]. In fact, VOCs are common subsurface contaminants found in urban groundwater systems across the USA [33,67]. Throughout the city of Detroit, VOCs are present at multiple brownfield sites and impact every neighborhood regardless of demographics (Figure 2 [68]).

**Figure 3:**
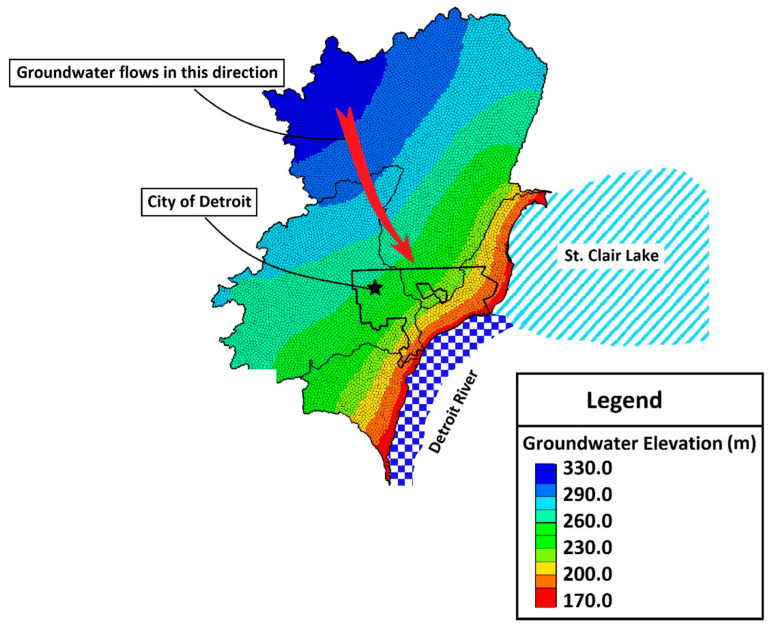
Regional flow features of shallow groundwater in Detroit. Flow rate is primarily towards the southeast.

**Figure 4:**
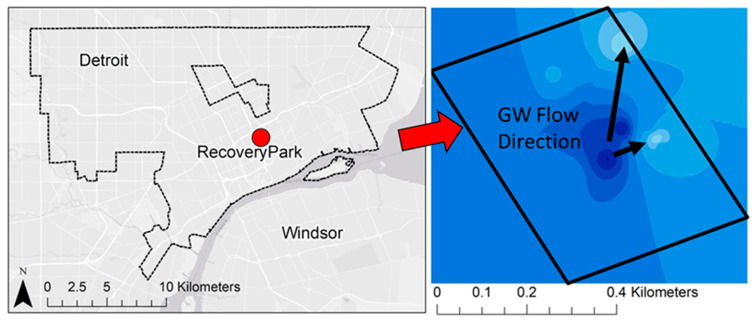
Local flow features of shallow groundwater in Recovery Park, Detroit. Flow rate is directed towards the north/northeast.

Though water is a critical resource in urban areas and groundwater accounts for between 22 and 42% of total water balance into the Great Lakes (with precipitation and surface water runoff accounting for the remainder) [69], urban groundwater flow in the coastal Great Lakes is poorly understood [70]. This is primarily because most urban centers rely on surface water as their water supply and underground piping networks to deliver fresh potable water. Thus, urban areas do not use groundwater, creating an ‘out of sight, out of mind’ mentality. Further, it can be difficult to characterize groundwater in these settings given the high degree of hydraulic and hydrologic modifications of the natural conditions [71]. Shallow urban subsurface systems, unlike natural systems, behave in unexpected ways requiring consideration of anthropogenic structures and alterations that can influence contaminant movement, including modified infiltration and percolation pathways due to surface disturbance and removal of vegetation, impervious/paved surfaces and roofs, sunken freeways, underground utility corridors, burial of demolition debris and artifacts (e.g., brick, coal slag, broken glass), transported fill soils, and mixing of soil material during excavation [72]. Over time, anthropogenic disturbance and manipulation of urban watersheds create unique human–hydrologic systems that connect the natural and built urban environments [60,61,73,74], resulting in multiple factors involved in assessing urban risk to environmental factors [75,76,77]. A simple example of this is provided by Figure 3 and Figure 4, which highlight the potential difference in transport inferences when moving from the regional to local scale, the latter of which is likely to show more anthropogenic distortion. Figure 3 shows the natural/regional groundwater flow in Detroit directed to the discharge point of the Detroit River, with the regional flow direction primarily towards the southeast. However at a local scale, this feature may be disturbed by local activities and the groundwater flow may be much different, as shown in Figure 4 for the local flows observed at Recovery Park (Detroit, MI, USA) with flow directed towards the north/northeast [78,79,80].

To increase the resiliency and sustainability of urban centers, anthropogenic changes to urban hydrology need consideration [81]. One way to consider these human–hydrologic systems is as ‘internal’ and ‘external’ water systems [82]. Internal systems denote drinking, sanitary, industrial, and heating piped water, while external systems denote precipitation, overland flow, infiltration, soil moisture, and groundwater. While these systems can be considered separate, many urban centers with old sewer systems have leakage along the supply pipes and sanitary pipes and/or have combined stormwater and sewer systems [82,83,84,85]. The high degree of modification of urban areas often creates environments controlled by anthropogenic modifications. An integrated urban water modeling approach can be employed to assess various components, as well as their interactions in human–hydrologic systems [71,74,86,87]. An urban water budget which uses a water mass balance for specified time and space can provide: (1) a framework to study the links between various elements of urban water [82,83,84,85,86,87,88]; (2) insight into the neighborhood or localized groundwater flow, and (3) a quantitative estimate for urban recharge [72]. Cities with a rich industrial legacy, such as Detroit, face many environmental concerns, including subsurface contamination [89]. Urban factors influencing VOC movement will be discussed in specific relation to Detroit.

The city of Detroit resides in a glacial depositional environment and on the historic glacial Lake Maumee depositional zone. This depositional environment is characteristic of large sediment layers, including large clay layers from the glacial lake period. The clay composition of subsurface sediments provides conditions for the formation of shallow groundwater [90,91,92,93]. The amount of water transferring through this shallow system is not well quantified since most of the water resource studies are focused on deep groundwater systems [93,94,95]. Guo et al. [66] noted that fluctuating groundwater tables present potential exposure routes via flooding and increasing proximity to groundwater. Evaluating vertical subsurface water movement can contribute to understanding this exposure route. Changes in groundwater storage impact groundwater movement, especially in shallow systems. Studies at Recovery Park (Detroit, MI, USA) field site showed hydrologic connection between the surface and shallow groundwater tables with a degree of interaction with wastewater conveyances and other inputs into aquifer [96]. There is evidence that VOCs can move through and be stored in soil profiles that include layering of different soil textures and structures [97,98]. VOCs are detected at high concentrations when released within clay units, but weakly absorb to particles in sandy soil texture with the potential to transfer considerable distances from the source [91,99].

Urban sewer lines represent another exposure pathway for VOC vapor intrusion into buildings [37,100,101]. This pathway is often neglected in vapor intrusion conceptual site models [42]. Utilities change the hydraulic conductivity of an area [76,77] by impacting both infiltration and horizontal movement [72], thus creating zones of preferential movement. The bedding material for the utilities also typically has a higher hydraulic conductivity than the surrounding media, creating a preferential pathway both horizontally and vertically. Similarly, disturbed sediments are more porous and permeable than naturally deposited sediments [102]. Conversely, fill material can create an ephemeral water table due to the contrasting permeability. These short-lived water tables can allow for horizontal fluid transport until there is sufficient head pressure to create vertical movement. In such a case, there is potential for a small point source pollutant to become an extensive shallow soil pollutant source [102]. This illustrates that understanding the subsurface utility influence on flow is an important component to developing a conceptual model of groundwater flow. The evaluation of preferential pathways as it applies to vapor intrusion is broad and can include natural features (e.g., sand lenses), building features (e.g., building footprint), and urban utilities (e.g., sewer pipes) [51].

The lack of groundwater data, continuing growth of urban areas, and pollution of urban groundwater create a potential threat to the long-term health and viability of the Great Lakes hydrologic system [70], pointing to the need for further groundwater research and integrated modeling approaches to inform proper management of VOC sources in urban settings.

## 3. Remediation Concepts Relevant to VOCs in Urban Settings

Remediation of VOC contamination can be generalized to three basic components for this review: (1) identifying the human exposure pathways, (2) identifying the geographic extent of contamination, and (3) minimizing the threat to human health by mitigating the exposure pathways. Possible human exposure pathways for VOCs related to brownfield sites are: (1) inhalation of indoor air impacted by vapor intrusion and (2) ingestion, dermal contact, and inhalation while showering with contaminated groundwater [103]. A remediation project begins with identifying the source and extent of contamination, exposure pathways, and in what phases the contaminant is moving downgradient. Since VOCs can move in the gas phase, in pure liquid phase (e.g., free-floating product), or dissolved in water, a remediation strategy must provide methods to address all of these potential transport modes. Remediation techniques can be distinguished as source zone remediation, which pertains to cleaning up contaminated sites and the associated VOC groundwater contaminant plumes, or vapor intrusion mitigation, which deals with the major symptom of subsurface VOC contamination, i.e., intrusion of VOC vapors into a building.

In general, the highest concentrations of free-floating product and dissolved concentrations are found at a contaminant source (e.g., gas station, dry cleaner, or auto repair shop). The dissolved concentration and volatilization potential are a function of the aqueous solubility (C^w^_sat_) and the air-water partitioning coefficient (*K_aw_* or *H*) of the contaminant [104]. Although elevated concentrations of VOCs in air can be measured in many urban environments [105], concentrations must exceed certain thresholds to require site/source remediation. A useful tool to link the possible connections between contaminant sources and human exposure (and thus the need for remediation) is a simplified diagram called a conceptual model. The U.S. Environmental Protection Agency (EPA) provides examples of VOC transport pathways and conceptual models for different site conditions [51,106]. Diffusion-dominated subsurface vapor transport models are used to inform remediation strategies by quantifying indoor air concentrations based on a source concentration, site characteristics (e.g., soil hydraulic properties, depth to groundwater, underground utilities), structural integrity, and contaminant physicochemical properties [106,107,108,109]. Subsurface heterogeneities such as soil layers, preferential pathways, and variability in moisture content have a strong impact on vapor transport [106]. In the unsaturated zone just beneath the surface, VOCs may be found as free product or vapor in soil pore space, dissolved in the porewater, adsorbed to soil grains, or floating above the water table in the case of light non-aqueous-phase liquids (LNAPLs) [110]. EPA has developed a vapor intrusion screening level calculator that can be used to determine screening and/or cleanup concentrations for groundwater, soil gas, and indoor air [51,111].

The regulatory framework for the modern environmental remediation industry in the U.S. is based on environmental regulations established in the 1970s and 1980s. Risk-based assessment tools were developed by EPA and others to correlate human exposure (quantified as a chronic daily intake—CDI) to risk. Acceptable risk from environmental chemical exposure is defined by EPA as an additional risk of mortality of 1 in 1,000,000. Remediation projects cannot proceed without an identified funding source, and litigation is often required to secure funds for remediation projects. In 1980, the U.S. Congress passed the Comprehensive Environmental Response, Compensation, and Liability Act (CERCLA) which created a tax on the petroleum and chemical industries to provide a financial means for conducting large-scale remediation projects on sites that “endanger public health or the environment” [112]. The framework that CERCLA established is often referred to as the Superfund. Zoning changes can also increase the value of a contaminated parcel of land to incentivize a site redevelopment project.

A remediation project typically begins in earnest with a Phase I Environmental Site Assessment (ESA) to investigate for a recognized environmental condition (REC). A Phase I ESA may be triggered due to real estate transactions, land use change applications, property owner concern, or when a regulatory agency has suspicion of contamination [113]. The purpose of Phase I includes four parts: records review, site reconnaissance, interviews, and a report on the environmental conditions on the property and data gaps that may be present [113].

If an REC is identified in Phase I, a Phase II ESA is commenced [114] and requires soil, groundwater, and building material sample collection for quantitative analysis [114]. The purpose of Phase II is to determine the geographic extent of contamination. Phase II remedial investigations may include (1) installation of soil borings and monitoring wells, (2) collection and analysis of soil and groundwater samples, and (3) pumping tests [110,115]. If a Phase II investigation identifies contamination, additional sampling and interpretation are required to delineate the geographic extent of the contamination. This may involve additional testing, contaminant transport modelling, remediation feasibility studies, and remediation plan development. Costs and alternative clean-up methods are also evaluated.

A suitable remediation strategy that reduces exposure to acceptable risk levels is developed based on site-specific conditions, clean-up criteria, costs, and the type of contaminated media (e.g., pore gas, water, and soil). Because surface water is the main drinking water source for most Great Lakes cities, and large treatment works and distribution systems are common, the main exposure pathways of interest for an urban risk-based remediation strategy are: (1) direct exposure (inhalation and dermal) from a contaminated site, and (2) possible vapor intrusion into buildings adjacent to the contaminated property. In this case, source zone remediation would reduce direct exposure, whereas site remediation would limit exposure to building occupants.

The three main source zone remediation approaches are: (1) isolation, (2) extraction, and (3) transformation [116]. Isolation techniques are used to physically or hydraulically separate the contaminant from the environment. Low permeability barriers made of steel, clay, and/or polymer sheets are used for physical containment [116,117], while networks of pumping wells can modify groundwater gradients to provide hydraulic isolation [98]. Extraction techniques include excavation of contaminated soil with earthmoving equipment for ex situ soil treatment or disposal [118], as well as integrated well and treatment systems called pump-and-treat systems [119], for which performance can be enhanced with solvents and surfactants [120]. Various methods of extracting multiphase mixtures of free product, porewater, soil vapor, and shallow groundwater have been developed. However, further treatment or disposal of the extracted material is required [121,122]. Extraction techniques produce large volumes of waste streams (e.g., air, water, and contaminated soil) that often require additional treatment. Due to the large volumes, removing VOCs below a certain threshold to allow release of air or water into the environment is the most economical approach. These techniques often rely on the use of sorbent materials such as activated carbon, zeolite, and organic polymers to remove VOCs from air and water [123,124,125]. An active area of research is the development and characterization of new sorbent materials that address shortcomings of traditional sorbents (e.g., humidity effects, performance goals above ppb ranges). One example is swellable organically modified silica, which has been used in applications ranging from sorbing dissolved aqueous species [126] to concentrating organic ligands for engineered resins [127,128] and has also been investigated as a potential air purifier sorbent [129,130]. Soil vapor extraction has also been used extensively [131].

Transformation remediation techniques provide methods for converting VOCs into smaller metabolites with the goal of complete mineralization. These techniques include chemical oxidation/reduction, thermal treatments, and various bioremediation strategies. Several mitigation techniques are often used in VOC remediation projects. Mitigation techniques eliminate or minimize the exposure pathway without eliminating the source from the environment. Increased building ventilation, sub-slab depressurization systems (SSDS), and sorbent-based air purifiers are notable techniques [116,132].

The selection of appropriate remedial technology depends on the nature of the contaminant. Non-halogenated or hydrocarbon VOCs are relatively biodegradable and can be treated through bioreactors, bioattenuation, biostimulation, and other related techniques. The Federal Remediation Technologies Roundtable (FRTR) evaluated 64 different potential remediation technologies for VOCs and noted widely used remediation strategies based on media are excavation (soil), pump and treat via carbon adsorption or air stripping (water), and soil vapor extraction (pore gas) [133]. Once a suitable remediation strategy has been developed, the remediation strategy is carried out by industry professionals. After a site is remediated, monitoring of air and water at site boundaries is conducted for a period of time to ensure the identified preliminary remediation goals (PRGs) have been met. Although there has been extensive research in many innovative and diverse VOC remediation techniques and technologies, the main challenge to application of new concepts is verification of predictable and reproducible results.

## 4. Health in Post-Industrial Cities and VOCs: A Focus on Birth Outcomes

Although sometimes criticized, the infant mortality rate has long been recognized as an important marker of a country’s health [134]. Low birth weight (birth weight less than 2500 g or approximately 5.5 pounds) and PTB (birth at less than 37 weeks gestation) are leading contributors to infant mortality [135] and therefore have immediate and lifelong health consequences including chronic diseases such as diabetes and cardiovascular disease [136,137,138]. Detroit currently has the highest PTB and infant mortality rates of all major cities in the U.S. [17], and may “rival areas of the Third World” [139]. In addition, both in Detroit and across the U.S., black women have higher rates of low birth weight and PTB infants [140]. Environmental factors are likely an important contributor to the adverse birth outcomes in Detroit and other post-industrial urban areas where there are significant racial disparities in both exposure to environmental contaminants [141,142] and adverse birth outcomes [140,143].

A small but growing number of studies have examined the associations between various VOCs and birth outcomes. However, results have been conflicting. In North Carolina, PCE exposure was estimated by residential proximity to PCE contamination; women with the highest PCE exposure (odds ratio (OR) = 1.3, 95% CI = 1.0–1.6) or women with a duration of PCE exposure of 11–20 weeks (OR = 1.3, 95% CI = 1.1–1.6) were more likely to deliver a PTB infant [13,144]. In a study in Cape Town, Massachusetts, births to women exposed to PCE-contaminated drinking water were compared to unexposed women (exposure data was defined based on pipe distribution maps and birth outcome data was obtained from birth records) [145]. PCE exposure was associated with younger gestational age at delivery (maximum 2 week difference in gestational age at delivery) and an increased risk of PTB (reported ORs between 1.1 and 1.9) [145]. In contrast, there was no association between VOC exposure, estimated based on tap water sampling from 75 towns, and preterm birth PTB in a New Jersey study [146] nor in a study of PCE or TCE exposure in New York women potentially exposed via industrial spills [12].

There is a growing body of evidence linking the mixture benzene, toluene, ethylbenzene and toluene (BTEX) or its individual components with adverse birth outcomes. In a study of pregnant women in the Infancia y Medio Ambiente (INMA) cohort in Spain, estimated exposure to benzene was calculated based on the mother’s residential address using ambient collection and land use regression. INMA women with estimated exposure to benzene levels >2.7 μg/m in the air across pregnancy had increased risk of PTB (OR = 6.46; 95% CI 1.58, 26.35) compared to women with lower benzene exposure levels [147]. Higher maternal benzene exposure, measured via a personal air sampler in the EDEN birth cohort study, was associated with lower birth weight (OR = 1.38; 95% CI = 1.03–1.84) [148]. In a large Canadian study of nearly 350,000 births from the Alberta Perinatal Database, xylene and toluene, which were estimated using spatial mining techniques, were each associated with greater odds of PTB (xylene OR = 1.08; 95% CI = 1.05, 1.12 and toluene OR = 1.14; 95% CI 1.11, 1.18) [149]. Using birth certificate and publicly available pollution data in Brazil, higher cumulative exposure in the 5 days before delivery to benzene (OR = 1.12; 95% CI = 1.01–1.23) and toluene (OR = 1.12; 95% CI = 1.01–1.23) were each associated with increased risk of PTB [16]. Data from our own study conducted in Detroit showed that higher ambient BTEX exposure was associated with increased risk of PTB (OR = 1.54; 95% CI 1.25, 1.89 per 5 unit increase in BTEX), even after adjusting for confounding factors including demographic and clinical characteristics, and neighborhood-level poverty [15].

Most of these studies, however, are limited by reliance on estimated exposure and thus are subject to bias due to potential exposure misclassification. Such misclassification may have contributed to the conflicting results reported for PCE and TCE and birth outcomes, but there is also a need to consider underlying social factors that clearly influence both exposure to environmental contaminants and pregnancy outcomes. There is a notable lack of data on how poverty, stress, racism, inadequate housing, and other social factors influence both susceptibility to adverse environmental exposures and their subsequent influence on birth outcomes [150,151]. Future studies that examine personal and biological levels of VOCs (especially mixtures) over pregnancy as well social factors and pregnancy outcomes are needed to better understand these relationships.

## 5. VOC-Induced Reproductive Health Outcomes in Animal Models

As PTB and other adverse birth outcomes are multifactorial pathological events, elucidating the role of VOC exposures in epidemiological studies alone is challenging. Animal models are useful tools to help establish a causative link between VOC exposure and adverse reproductive and developmental outcomes due to more stringent control over additional environmental stressors and genetic variability. Toxicologists utilize animal models in which dose-response relationships can be more easily manipulated and controlled compared to human epidemiological studies to informing mechanisms of toxicity and intervention strategies. Data from both non-mammalian and mammalian models further supports a link between VOCs and reproductive and development health.

The reproductive and early developmental effects of VOC exposure have been explored in a variety of non-mammalian models. Benzene or toluene exposure negatively affects embryonic growth in fathead minnows (*Pimephales promelas*) and African sharptooth catfish (*Clarias gariepinus*) in a dose-dependent manner [152,153]. Additionally, toluene exposure results in developmental abnormalities in fathead minnows and Japanese medaka (*Oryzias latipes*) related to neurological, cardiovascular, and/or ocular development, as well as distortion of the embryonic axis [154,155]. Similar abnormalities, as well as significantly decreased larval weight and length, were observed in medaka and zebrafish (*Danio rerio*) after PCE and TCE exposure, respectively [156,157,158,159,160]. Medaka exposed to toluene at various stages of development also had significant, dose-dependent delays in hatching times and reductions in percentage of successfully hatched eggs [155]. Similarly, fruit fly (*Drosophila melanogaster*) larvae exposed to benzene, toluene, or ethylbenzene throughout development showed a dose-dependent delay in emergence from the pupa and decrease in overall emergence that were associated with significant increases in expression of common stress genes (e.g., hsp70, hsp60, hsp83, and hsp26) and generation of reactive oxygen species [161]. These findings were subsequently described as showing a significant link between reproductive success and oxidative stress [162]. Similarly, a study in zebrafish demonstrated TCE-induced changes in gene expression involved in oxidative stress pathways [157], while exposing fruit flies to benzene, toluene, and/or xylene resulted in significant increases in generation of reactive oxygen species and induction of oxidative stress biomarkers [163]. These non-mammalian studies reveal the potential for impacts on early life growth and mortality, as well as abnormalities that affect numerous vital physiological and anatomic systems, due to VOC exposure at developmental stages. The connections between these outcomes with oxidative stress and immune function deserve more thorough investigation in future studies.

In mammalian models, there is also evidence for a role of VOCs in adverse birth outcomes. Perhaps the most commonly observed adverse birth outcome in rodents related to maternal VOC exposure is decreased fetal growth. Much work has focused on exposures related to benzene, toluene, ethylbenzene and xylene (BTEX), but some evidence related to other VOCs such as TCE also exists in mammalian systems [164]. In chronic exposure studies, which mimic the conditions of occupational or at home exposure [165], pregnant rodents exposed to toluene via whole-body inhalation chamber at concentrations of 1000–1800 ppm for 6 h per day had offspring with significantly reduced body weight (~10% decrease) which has been shown to remain lower for at least 2 weeks after birth [166,167,168]. In addition, low level prenatal exposure to toluene, xylene, or benzene (<400 ppm) for 24 h per day resulted in decreased average fetal weights in rats and mice compared to pure air controls [169]. In this same study, researchers observed ~40% of offspring from exposed dams weighing less than 3.3 g (rats) or 0.9 g (mice). However, not all chronic prenatal exposure studies resulted in significant reductions in offspring birthweight. In one study, in which young female rats were exposed to 500 ppm xylene for 6 h per day via whole-body inhalation chamber, birthweight differences were not statistically significant [170]. Furthermore, a recent study by Malloul et al. [171] via whole-body inhalation chamber to paint thinner at 300 or 600 ppm [chemical composition most represented by toluene (24.46%), xylene (15.47%), benzene (10.67%), dichloromethylene (6.34%) and acetone (5.55%)] revealed that exposure did not significantly affect birth weight at postnatal day (PND) 1. However, over the subsequent 3 weeks, mice exposed to paint thinner gained significantly less weight (i.e., ~20% less) at postnatal days 14 and 21 [171]. In animal models designed to mimic acute high exposures, rodents exposed to repeated sessions of high concentrations of toluene or 1,1,1-trichloroethane (2000–12,000 ppm) displayed either decreased weight at birth or decreased weight gain over the next few weeks [172,173]. In addition to overall fetal growth, congenital heart defects (CHDs) have been observed as an adverse birth outcome in rodent models. The fetotoxic effects of toluene and other BTEX chemicals have been demonstrated in controlled studies in animals and in some cases mirror what is seen in human epidemiological studies. For example, one study reported increases in total malformations at doses of xylene beginning at 2.06 mg/kg per day, with cleft palate being the most common disorder (bilateral open eye, exencephaly, and fused or missing vertebral arches and ribs were also observed) [174]. Interestingly, this study chose to gavage pregnant mice the xylene mixture dissolved in oil on days 6–15 of gestation instead of the more commonly used inhalation exposure methods.

Rodent models have also been utilized to identify mammalian toxicokinetics, dynamics, and metabolism of benzene and related VOCs during pregnancy. Pregnant women mostly share the same distribution processes as other non-pregnant adults, but pregnancy-related weight gain, differential perfusion of tissues and fat, and the formation of the placenta as a new tissue compartment may modulate the kinetics and distribution of BTEX chemicals [175]. By using radiolabeled benzene, toluene, and xylene, Ghantous and Danielsson [176] discovered rapid elimination of these chemicals in well perfused organs in pregnant mice, but retention was observed in adipose tissue, liver, and kidney. BTEX-type chemicals can cross the placenta directly impacting gestation [177]. Additionally, retention of radioactive metabolites in placenta was seen 1 h after inhalation exposure. In another study, the blood level of toluene in pregnant rats (2.1 mg%) was observed to be higher than in the virgin counterparts (1.0 mg%) [178]. Importantly, a higher peak blood concentration and area under blood concentration vs. time curve (AUC) is also predicted in the human acute exposure physiologically based pharmacokinetic (PBPK) model for benzene inhalation during pregnancy [179]. In addition to differences in distribution of VOCs, metabolism of these compounds may also differ during pregnancy. Absorbed BTEX-like chemicals are metabolized by liver cytochrome P450 enzymes, primarily CYP2E1 [180]. In the case of benzene, endogenous metabolism to reactive intermediates appears to be a primary mediator of toxicity [181]. Importantly, animal studies indicated that expression of CYP2E1 significantly decreases in mouse and rat liver during pregnancy [182,183]. It appears that BTEX metabolism is negligible within the fetal liver as expression of CYP2E1 has not been detected in mammalian fetal liver samples [184]. Finally, singular exposure to one of the components of BTEX is rare. Therefore, future mechanistic studies should investigate the impact of VOC mixtures on metabolism and health outcomes in a pregnancy setting. Current data is lacking, but through modeling approaches, Haddad et al. [185] suggests competitive inhibition of the cytochrome P450 system is a possible scenario when considering the toxic effects of a tertiary BTEX mixture [185].

Emerging data now implicates benzene, toluene, ethylbenzene, xylene, and related VOC exposures during pregnancy on disruptions to the fetal immune system. The use of animal models is useful to link VOC exposure with disruptions of the fetal immune system, but mechanistic studies using well-established rodent models are currently lacking and require more attention. In one such example, however, pregnant mice were injected with benzene (100 mg/kg, twice daily) from day 12.5 through 19.5 of gestation [186]. The researchers observed fewer pre-B-lymphocyte cells and fewer mature B cells in offspring from exposed mothers compared to corresponding controls. Additionally, mice born to exposed mothers displayed decreased responsiveness to lipopolysaccharide (LPS). In conclusion, the growing development and utilization of animal models to explore the reproductive and early developmental effects of VOC exposure have helped to elucidate a link to adverse health effects as well as possible mechanisms of toxicity. Further animal studies will be crucial for the development of potential human intervention strategies and therapies for VOC exposures.

## 6. Conclusions

Belowground VOCs and the related vapor intrusion process are understudied despite evidence that these contaminants are widespread beyond Superfund and brownfield sites, as well as epidemiologic and animal model studies suggesting significant adverse birth outcomes and early life health consequences following exposure to VOCs that are commonly found indoors. The COVID-19 pandemic has served to further add relevance and urgency due to increased time spent indoors. In order to better understand volatile and semi-volatile organic compounds, specifically in urban environments, future research must: (1) focus on groundwater data and integrated modeling that accounts for the high degree of anthropogenic hydraulic and hydrologic modifications affecting subsurface VOC movement; (2) identify VOC remediation techniques and technologies that produce predictable and reproducible results; (3) examine personal and biological levels of VOCs and VOC mixtures throughout pregnancy, underlying social factors that influence exposure, and subsequent health outcomes; and (4) develop and utilize animal models to elucidate a link between environmentally-relevant VOC exposure and reproductive and early developmental effects, as well as mechanisms of toxicity, with the goal of informing and driving epidemiologic studies and developing human intervention strategies and therapies. Addressing these knowledge gaps will require robust transdisciplinary collaboration and is essential for not only tackling health disparities and environmental injustice in post-industrial cities such as Detroit, but also protecting and preserving critical freshwater resources within the Great Lakes basin.
